# Placement of Covered Self-Expandable Metal Biliary Stent for the Treatment of Severe Postsphincterotomy Bleeding: Outcomes of Two Cases

**DOI:** 10.1155/2010/138748

**Published:** 2010-06-06

**Authors:** Marta Di Pisa, Ilaria Tarantino, Luca Barresi, Davide Cintorino, Mario Traina

**Affiliations:** Gastroenterology Service, IsMeTT, UPMC, Via Tricomi 1, 90146 Palermo, Italy

## Abstract

We report two cases of severe postsphincterotomy bleeding in an adult and a pediatric patient treated, as first options, with available techniques to induce hemostasis without success. Because of persisting bleeding, an expandable, partially covered, metallic stent was placed into the choledocho to mechanically compress the bleeding site. The bleeding was stopped. In the following days, both patients remained hemodynamically stable with no further episodes of bleeding. We believe that the application of a covered metallic stent in a severe postendoscopic-sphincterotomy bleeding, refractory to injection therapy, should be considered to avoid additional interventions, which carry a higher risk of complications, even in pediatric patients.

## 1. Introduction

 Endoscopic retrograde cholangiopancreatography (ERCP) has become an important therapeutic procedure for a great variety of biliary and pancreatic diseases [1]. Sphincterotomy is used to remove biliary and pancreatic stones, to facilitate placement of biliary and pancreatic stents, and to treat patients with sphincter-of-Oddi dysfunction [2]. One of the most frequent complications of sphincterotomy is bleeding [3, 4]. We report two cases of use of a covered metallic stent to treat severe postsphincterotomy bleeding.

## 2. Case Report 1

**Figure 1:**
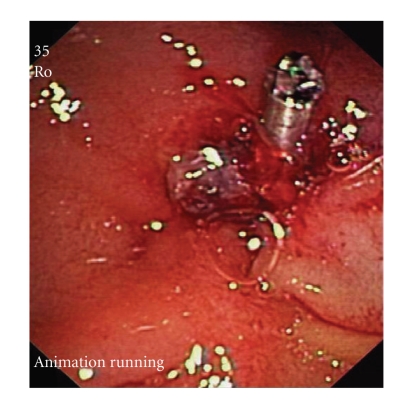
A clip was placed at the site of the sphincterotomy.

**Figure 2:**
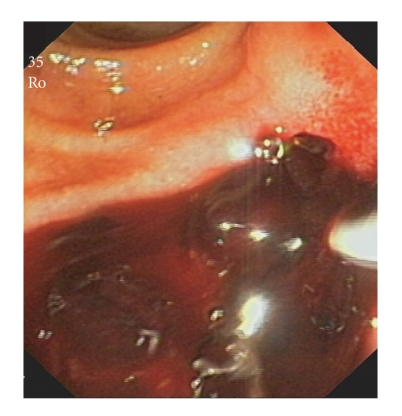
Severe bleeding from the sphincterotomy site.

**Figure 3:**
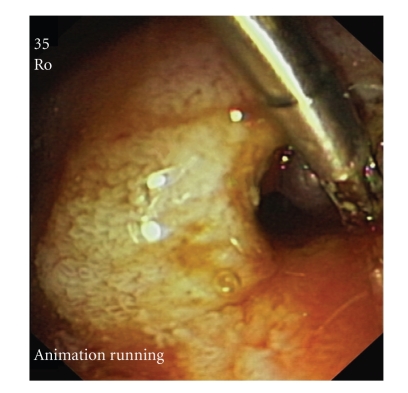
After clip placement and sclerosis, a vessel behind the clip is visible (arrow).

**Figure 4:**
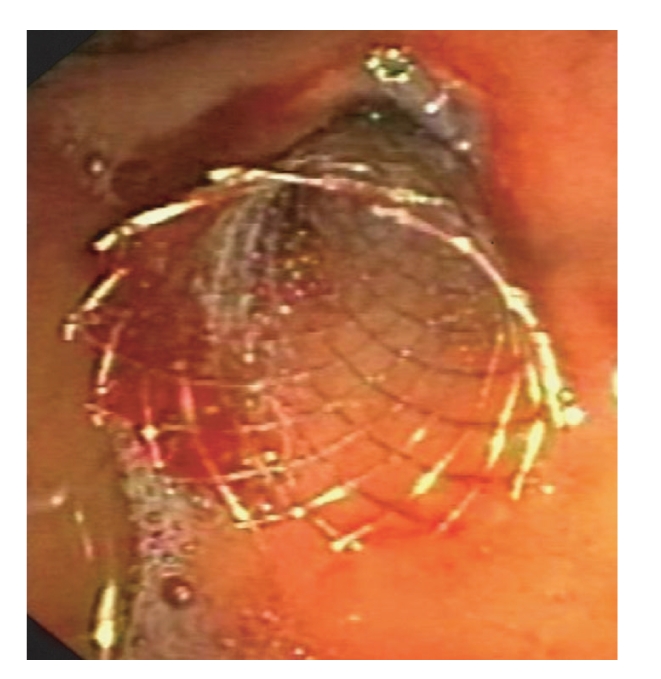
The metallic stent is placed.

**Figure 5:**
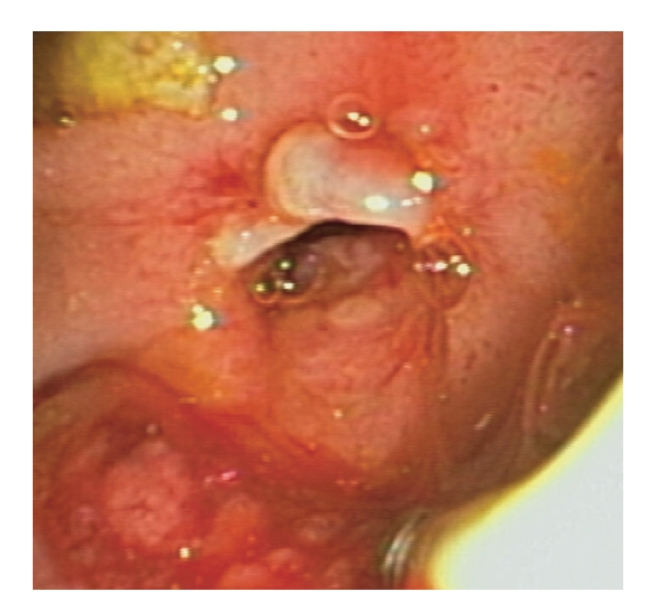
No signs of bleeding after metallic stent removal.

 A 52-year-old man underwent laparoscopic cholecystectomy in 2002. Since then, the patient has had recurrent episodes of abdominal pain and fever. An ERCP was performed in 2004, with sphincterotomy and sludge removal. Another episode of cholangitis led to an additional procedure, performed in March 2005, with pneumatic and mechanical dilation of the sphincterotomy and sludge removal. The patient has since experienced other episodes of cholangitis, treated with antibiotic therapy only, the last one in July 2006, with evidence of cholestasis and choledochus dilation at sonography. For this reason, the patient was admitted to our institute to undergo ERCP. The patient was in good general condition, and physical examination was unremarkable. Laboratory data were Hb 15.3 gr/dL, HCT 44%, direct bilirubin 0.68 mg% gammaglutamyl transferase 281 U/L, AST/ALT 42/186 U/L, and alkaline phosphates 146 U/L. We found no comorbidities. On piperacillin/tazobactam, the patient underwent ERCP, showing mild dilation of the choledochus with several filling defects. The sphincterotomy was enlarged with a sphincterotome on a guide wire (endocut: watt 120; coag: watt 40, PSD 60 Olympus) and biliary sludge was removed with a Fogarty balloon catheter. The procedure was well tolerated and no signs of bleeding were detected. Almost 6 hours after the procedure, the patient had an episode of rectal bleeding. Blood tests showed Hb 13.3 gr/dL, HCT 39.3%, 120/80 mmHg, and heart rate 70/min. As a result, an emergency esophageal gastroduodenoscopy (EGD) was performed. It showed active bleeding from the site of previous sphincterotomy. Adrenaline was injected (1 : 10.000 dilution, 10 mL) and a metallic clip (Resolution Clip, Boston Scientific Corporation, Natik USA) was placed, with an evident cessation of hemorrhaging (Figure 1). The following day, the patient had two episodes of severe rectal bleeding. Hemoglobin levels were stable at 11.7 gr/dL, Hct 35%, arterial blood pressure was 120/70 mmHg, and heart rate was 70/min. Another EGD was performed and showed a persistent and severe bleeding from the previous clip placement site (Figure 2). Sclerosis with adrenaline (1 : 10.000 dilution, 10 mL) was redone, and a plastic stent (10 Fr, 5 cm, OASIS, Wilson Cook Medical, Winston-Salem, NC) was placed, with subsequent cessation of the bleeding. Almost 3 hours after the sclerotherapy, the patient became hemodynamically instable, hypotensive, and tachicardic, with drop in hemoglobin level (Hb: 4 gr/dL). Another episode of hematochezia occurred. EGD was repeated once more and showed a large amount of blood and clots in the stomach, and fresh bleeding from the sphincterotomy. The plastic stent was removed with a snare, and multiple clots were removed from the biliary duct with a balloon catheter, exposing a small, nonbleeding vessel in the site of the sphincterotomy (Figure 3). Severe bleeding occurred. In order to stop the bleeding, an 8 mm dilation balloon catheter (8 mm × 2 cm Hurricane Microvasive, Boston Scientific Corporation, Natik USA) was used to tampon the hemorrhage temporarily. But because of persisting of the bleeding from the sphincterotomy site when the balloon was removed, an expandable, partially covered metallic stent (4 cm in length and 1 cm in diameter, Wallstent, Boston Scientific Corporation, Natik USA) was placed in the choledocho to mechanically compress the bleeding site and drain the clots from the biliary duct (Figure 4). The patient was admitted to the intensive care unit. After volume replacement (almost 2 L) and transfusion of 4 units of packed red blood cells, the patient stabilized hemodynamically (Hb: 8 gr/dL). On postprocedure day 5, the patient was discharged home in good clinical condition. We saw him one month later in the outpatient clinic. He was in good general condition, asymptomatic, and with normal blood tests. An ERCP was scheduled to remove the previously placed metallic stent. The ERCP showed a partial obstruction in the metallic stent and was removed with a snare. The cholangiography showed multiple filling defects in the upper third of the choledocho. No signs of bleeding were seen (Figure 5). A Fogarty balloon catheter was used to remove the sludge. The patient was discharged home the day after the procedure in good general conditions, asymptomatic, and with normal blood tests. After 8 months, the patient is still asymptomatic and in good general conditions.

## 3. Case Report 2

**Figure 6:**
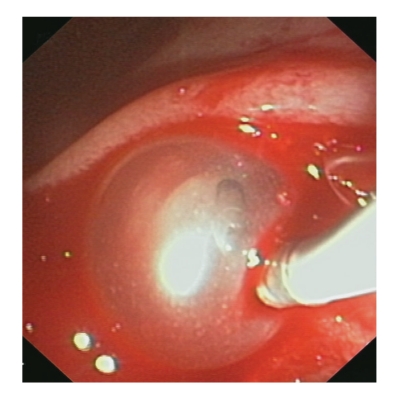
A stone extraction balloon cathether was used for compression.

**Figure 7:**
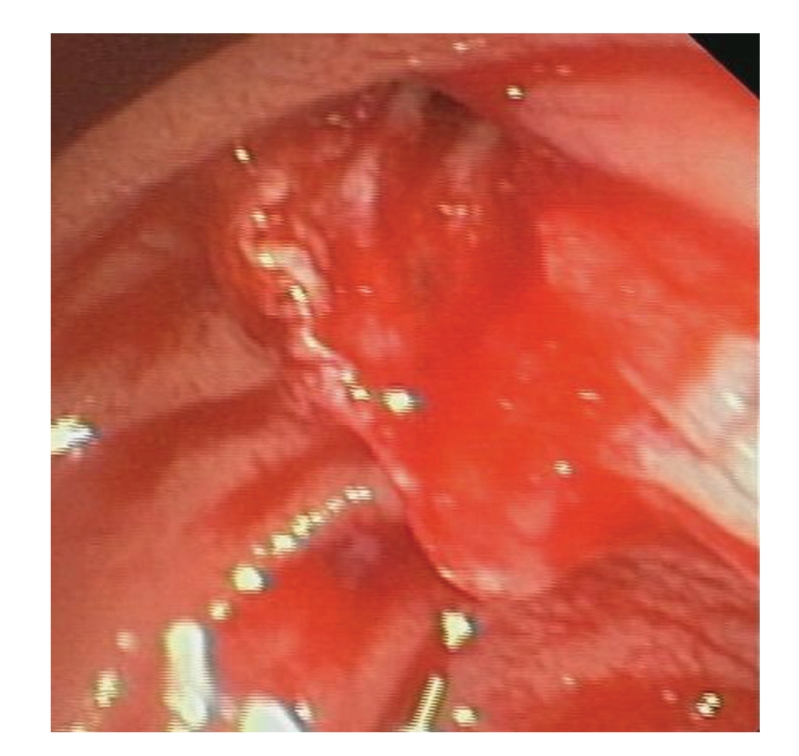
Severe bleeding from the upper margin of the sphincterotomy site.

**Figure 8:**
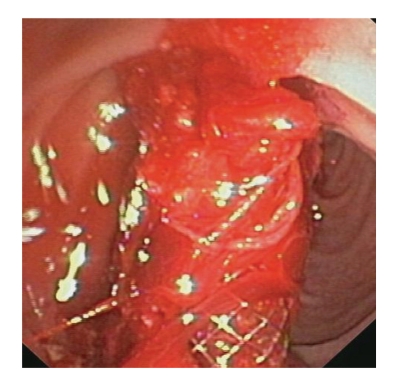
Expandable covered metallic stent placement.

**Figure 9:**
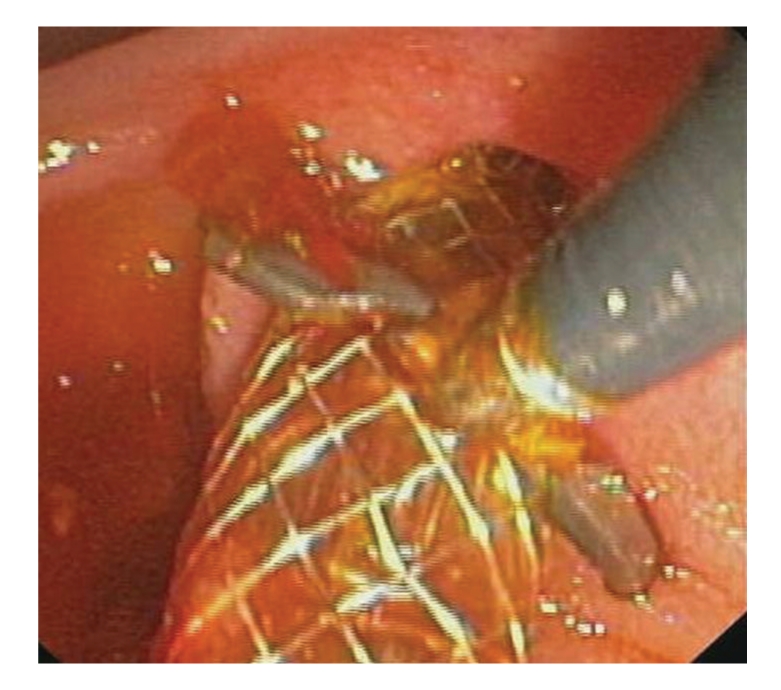
The metal stent is removed with alligator-tooth forceps.

**Figure 10:**
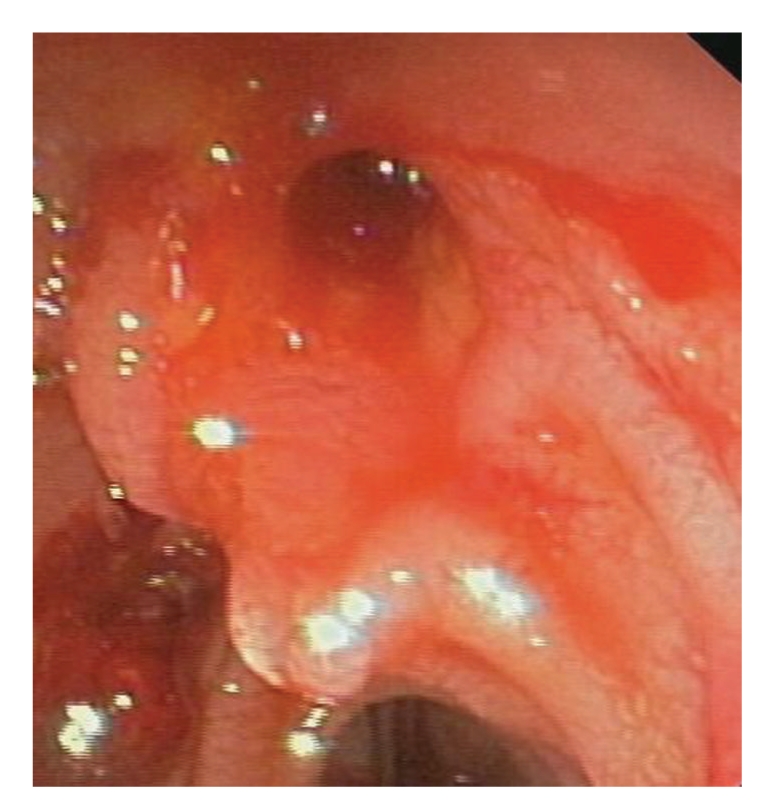
No signs of bleeding after stent removal.

 An 8-year-old boy was admitted to our institute because of portal cavernoma, perhaps secondary to umbilical vessels that were cannulated for neonatal sepsis, and esophageal varices were treated with two sessions of banding ligation. Another comorbidity found was congenital combined immunodeficiency (SCID T-B+). During followup, a CT scan, performed in order to evaluate his vascular anatomy for a possible surgical shunt, showed a 6.5 cm hepatic fluid lesion of unknown origin with a suspected stone inside, mild intrahepatic biliary duct dilation, and a large cavernoma of the portal vein. Laboratory data were AST/ALT 42/74 U/L (normal: 5–40/65 U/L), bilirubin tot/dir 0.49/0.11 mg/dL (0–1.5 mg/dL), alkaline phosphates 362 U/L (40–134 U/L), gamma-GT 70 mg/(5–85 U/L), PLT 60 10  *μ*L (50–400 10 *μ*L), PT 84%  (80%–120%), and INR 1.16 (0.80–1.20 INR). With evidence of cholestasisand a CT scan that prompted suspicion of a biliary stone, an ERCP was planned. At admission, the patient was in good general conditions, and the physical examination was unremarkable (weight Kg 20, arterial blood pressure 86/52, and heart rate 84). On piperacillin/tazobactam and under general anesthesia, the patient, continuously monitored with electrocardiography, pulse oximeter, and automatic recording of blood pressure and pulse, underwent ERCP, showing hilar extrinsic compression, with several filling defects. The sphincterotomy was done with a sphincterotome on a guide wire (endocut: watt 120; coag: watt 40, PSD 60 Olympus). Severe bleeding occurred. The lumen was filled entirely with blood, and only after ten minutes of washout and compression with an 8mm dilation balloon catheter (8 mm × 2 cm Hurricane Microvasive, Boston Scientific Corporation, Natik USA) and then with a stone extraction balloon catheter (15 mm, Extractor XL, Boston Scientific Corporation, Natik USA) (Figure 6), the bleeding site was seen in the upper margin of the sphincterotomy (Figure 7). Several attempts at sclerosis with 1 : 10.000 epinephrine, total 20 mL, were injected without stopping the bleeding. The patient became hypotensive, and hemoglobin level dropped to 7 gr/dL (from 10.4 gr/dL). We immediately started volume replacement (almost 1 L) and transfusion of 450 cc of packed red blood cells. Given our previous experience with a metal stent in post-sphincterotomy bleeding in an adult patient, we decided, 90 minutes into the procedure, to insert an expandable, covered metallic stent (4 cm in length and 1 cm in diameter, Wallstent, Boston Scientific Corporation, Natik USA) into the choledocho to mechanically compress the bleeding site and simultaneously drain the biliary duct (Figure 8). The bleeding ceased and the patient was admitted to the intensive care unit, in hemodynamically stable condition (Hb: 8.3 gr/dL). On post-procedure day 5, the patient was discharged home in good clinical conditions. Two weeks later, he underwent an open procedure to construct a mesocaval shunt. He tolerated this well, and 3 days later an ERCP to remove the stent was planned. Under general anesthesia, a lateral view endoscope was introduced and advanced to the papilla, where the stent was seen. The stent was patent and was removed with alligator-tooth forceps (Figure 9). The cholangiogram was normal, and no signs of bleeding were seen (Figure 10). Now, at six months after the procedure, the patient is in good general conditions, asymptomatic, and without signs of cholestasis.

## 4. Discussion

 Generally, postsphincterotomy bleeding is common (2%–11%) and self-limiting. Irrigation with sclerosants is usually sufficient to stop the bleeding [3, 4]. Other modalities include endoscopic balloon tamponade [5], thermal therapy with argon plasma coagulation [6], use of fibrin glue, and clip placement [7]. For refractory cases, angiographic embolization, or surgery, is necessary [8]. Several studies have shown that serial placement of biliary stents of incrementally increasing diameter is successful in the treatment of post-sphincterotomy stenosis. Moreover, a recent case report documented the successful use of a covered metallic stent in active bleeding from esophageal varices [9]. This evidence in the literature prompted us to apply the stent in an attempt to stop the bleeding. We used the compressive effect of the stent in the first case. In the second case, because we had successfully used the stent in an adult patient to treat post-sphincterotomy bleeding, and because patient was young, we opted to forego the other available methods. The main indication for the use of metal stents is biliary obstruction [10]. Recently, use of these stents has progressively expanded to treatment of other pathologic conditions [11, 12]. Placement and removal of these stents are easy, though removal must be done within no more than 3 months. In the first case, we attempted everything but embolization to stop the bleeding. Moving the patient (intubated and in hypotensive condition) to the angiographic room was too difficult and, indeed, dangerous. In the second case, we relied on our previous experience in an adult patient, using a metal stent to stop the hemorrhaging. In conclusion, we believe that in persistent post-ES bleeding, when refractory to injection therapy or when not all modalities to induce hemostasis are available, the application of a covered metallic stent should be considered a further therapeutic option, allowing the endoscopist to avoid additional procedures, with higher risks of complications, especially in very young patients, who could have more serious complications. However, further studies and new advanced metallic prostheses with adequate features (longer length, great bile resistance, and easy removal) are needed to ensure safe and efficient use.

## References

[1] Costamagna G (2000). Therapeutic biliary endoscopy. *Endoscopy*.

[2] Sherman S, Ruffolo TA, Hawes RH, Lehman GA (1991). Complications of endoscopic sphincterotomy: a prospective series with emphasis on the increased risk associated with sphincter of Oddi dysfunction and nondilated bile ducts. *Gastroenterology*.

[3] Cotton PB, Lehman G, Vennes J (1991). Endoscopic sphincterotomy complications and their management: an attempt at consensus. *Gastrointestinal Endoscopy*.

[4] Leung JWC, Chan FKL, Sung JJY, Chung SCS (1995). Endoscopic sphincterotomy-induced hemorrhage: a study of risk factors and the role of epinephrine injection. *Gastrointestinal Endoscopy*.

[5] Staritz M, Ewe K, Goerg K, Meyer zum Büschenfelde KH (1984). Endoscopic balloon tamponade for conservative management of severe hemorrhage following endoscopic sphincterotomy. *Zeitschrift für Gastroenterologie*.

[6] Oviedo JA, Barrison A, Lichtenstein DR (2003). Endoscopic argon plasma coagulation for refractory postsphincterotomy bleeding: report of two cases. *Gastrointestinal Endoscopy*.

[7] Baron TH, Norton ID, Herman L (2000). Endoscopic hemoclip placement for postsphincterotomy bleeding. *Gastrointestinal Endoscopy*.

[8] Ferreira LEVVC, Baron TH (2007). Post-sphincterotomy bleeding: who, what, when, and how. *American Journal of Gastroenterology*.

[9] Hubmann R, Bodlaj G, Czompo M (2006). The use of self-expanding metal stents to treat acute esophageal variceal bleeding. *Endoscopy*.

[10] Suk KT, Kim JW, Kim HS (2007). Human application of a metallic stent covered with a paclitaxel-incorporated membrane for malignant biliary obstruction: multicenter pilot study. *Gastrointestinal Endoscopy*.

[11] Kahaleh M, Behm B, Clarke BW (2008). Temporary placement of covered self-expandable metal stents in benign biliary strictures: a new paradigm? (with video). *Gastrointestinal Endoscopy*.

[12] Wasan SM, Ross WA, Staerkel GA, Lee JH (2005). Use of expandable metallic biliary stents in resectable pancreatic cancer. *American Journal of Gastroenterology*.

